# What Is the “Optimal” Target Mismatch Criteria for Acute Ischemic Stroke?

**DOI:** 10.3389/fneur.2020.590766

**Published:** 2021-01-13

**Authors:** Chushuang Chen, Mark W. Parsons, Christopher R. Levi, Neil J. Spratt, Longting Lin, Timothy Kleinig, Kenneth Butcher, Xin Cheng, Qiang Dong, Billy O'Brien, Richard I. Avivi, Martin Krause, P. N. Sylaja, Philip Choi, Sandeep Bhuta, Congguo Yin, Jianhong Yang, Peng Wang, Weiwen Qiu, Andrew Bivard

**Affiliations:** ^1^Melbourne Brain Centre, University of Melbourne, Parkville, VIC, Australia; ^2^Department of Neurology, Liverpool Hospital, University of New South Wales, Sydney, NSW, Australia; ^3^Department of Neurology, John Hunter Hospital, University of Newcastle, Callaghan, NSW, Australia; ^4^Hunter Medical Research Institute, New Lambton Heights, NSW, Australia; ^5^Department of Neurology, Royal Adelaide Hospital, Adelaide, SA, Australia; ^6^Prince of Wales Medical School, University of New South Wales, Sydney, NSW, Australia; ^7^Department of Neurology, Huashan Hospital, Fudan University, Shanghai, China; ^8^Department of Neurology, Gosford Hospital, Gosford, NSW, Australia; ^9^Division of Neuroradiology, Department of Medical Imaging, University of Toronto and Sunnybrook Health Sciences Centre, Toronto, ON, Canada; ^10^Department of Neurology, Kolling Institute, Royal North Shore Hospital, University of Sydney, Sydney, NSW, Australia; ^11^Department of Neurology, Sri Chitra Tirunal Institute for Medical Sciences and Technology, Thiruvananthapuram, India; ^12^Department of Neurology, Box Hill Hospital, Melbourne, VIC, Australia; ^13^Department of Medical Imaging, Gold Coast University Hospital, Southport, QLD, Australia; ^14^Department of Neurology, Hangzhou First Hospital, Zhejiang, China; ^15^Department of Neurology, Ningbo First Hospital, Zhejiang, China; ^16^Department of Neurology, Taizhou First People's Hospital, Zhejiang, China; ^17^Department of Neurology, Lishui People's Hospital, Zhejiang, China

**Keywords:** ischemic stroke, perfusion, target mismatch, intravenous thrombolysis, endovascular thrombectomy

## Abstract

We aimed to compare Perfusion Imaging Mismatch (PIM) and Clinical Core Mismatch (CCM) criteria in ischemic stroke patients to identify the effect of these criteria on selected patient population characteristics and clinical outcomes. Patients from the INternational Stroke Perfusion Imaging REgistry (INSPIRE) who received reperfusion therapy, had pre-treatment multimodal CT, 24-h imaging, and 3 month outcomes were analyzed. Patients were divided into 3 cohorts: endovascular thrombectomy (EVT), intravenous thrombolysis alone with large vessel occlusion (IVT-LVO), and intravenous thrombolysis alone without LVO (IVT-nonLVO). Patients were classified using 6 separate mismatch criteria: PIM-using 3 different measures to define the perfusion deficit (Delay Time, Tmax, or Mean Transit Time); or CCM-mismatch between age-adjusted National Institutes of Health Stroke Scale and CT Perfusion core, defined as relative cerebral blood flow <30% within the perfusion deficit defined in three ways (as above). We assessed the eligibility rate for each mismatch criterion and its ability to identify patients likely to respond to treatment. There were 994 patients eligible for this study. PIM with delay time (PIM-DT) had the highest inclusion rate for both EVT (82.7%) and IVT-LVO (79.5%) cohorts. In PIM positive patients who received EVT, recanalization was strongly associated with achieving an excellent outcome at 90-days (e.g., PIM-DT: mRS 0-1, adjusted OR 4.27, *P* = 0.005), whereas there was no such association between reperfusion and an excellent outcome with any of the CCM criteria (all *p* > 0.05). Notably, in IVT-LVO cohort, 58.2% of the PIM-DT positive patients achieved an excellent outcome compared with 31.0% in non-mismatch patients following successful recanalization (*P* = 0.006).

**Conclusion:** PIM-DT was the optimal mismatch criterion in large vessel occlusion patients, combining a high eligibility rate with better clinical response to reperfusion. No mismatch criterion was useful to identify patients who are most likely response to reperfusion in non-large vessel occlusion patients.

## Introduction

Selection of patients using target mismatch can identify acute ischemic stroke patients who are most likely to benefit from intravenous thrombolysis (IVT) or endovascular thrombectomy (EVT) in an extended time window (1–3). However, the exact patient selection criteria remain a controversial topic. The DEFUSE3 (3) and EXTEND IA (4) using Perfusion Imaging Mismatch (PIM), which preferentially enroll patients with a largely treatable penumbra and small ischemic core. The DAWN trial (1) applied a Clinical-Core Mismatch (CCM) where an age-adjusted National Institutes of Health Stroke Scale (NIHSS) score was used as a surrogate for the total perfusion deficit, in combination with a small age-adjusted ischemic core define mismatch. However, various thresholds calculated by different post-processing algorithms, defining penumbra and core, has been reported. The most common set of thresholds defining penumbra and core are time to peak of the residual function (Tmax) > 6 s and relative cerebral blood flow (rCBF) <30%, or delay time (DT) >3 s and rCBF <30% (5). When calculating Mean Transit Time (MTT), Tmax and CBF by singular value deconvolution (sSVD), the algorithm assumes no delay in blood flow from proximal arteries to the ischemic region, as, almost invariably in ischemic stroke, there is delay and dispersion of the contrast between the more proximal arterial input function (AIF) and the ischemic region (6). The sSVD is a delay-sensitive algorithm, resulting in underestimation of CBF and overestimation of MTT (6–8). This is highly clinically relevant as different definitions of the perfusion deficit may affect reperfusion treatment eligibility. It is a challenge to determine which mismatch criteria are superior to others in term of optimally identifying excellent reperfusion responders and excluding those who are either likely to be harmed or who have a good natural history regardless of treatment, in routine clinical practice.

Therefore, in this study we aimed to: (i) to compare the various PIM and CCM criteria using different definitions of perfusion deficit; and (ii) assess the ability of each criterion to identify acute stroke patients who are most likely to respond to reperfusion treatment in different subgroups of acute ischemic stroke patients. We hypothesized: (i) that there would be considerable differences in the proportion of patients selected with each mismatch criterion; and (ii) that the presence of PIM or CCM positivity may not uniformly predict response to reperfusion treatment in different sub-groups of acute ischemic stroke patients.

## Methods

### Patients

Consecutive acute ischemic stroke patients presenting to 14 centres between 2012 and 2017 were prospectively recruited into the INternational Stroke Perfusion Imaging REgistry (INSPIRE). From the INSPIRE database, patients with anterior circulation ischemic stroke were included in this study if they fulfilled the following criteria:

- Received reperfusion therapy: Endovascular Thrombectomy (EVT) or Intravenous Thrombolysis (IVT) based on institutional guidelines.
- Underwent pre-treatment multimodal CT including non-contrast CT, CTP, CT angiography (CTA).
- Underwent 24-h imaging with MRI or multimodal CT.

Stroke severity was assessed at baseline and 24-h using NIHSS. Functional outcome was assessed at day-90 using the modified Rankin Scale (mRS). Patients were divided into two binary outcomes: excellent clinical outcome (mRS of 0-1 VS. mRS of 2-6), and good clinical outcome (mRS of 0-2 VS. mRS of 3-6). Symptomatic Intracranial Hemorrhage (sICH) was defined as type 2 parenchymal haematoma on follow-up imaging with more than 4-point increase in NIHSS or leading to death (9).

#### Patient Cohorts

Patients were divided into 3 cohorts. Cohort A (EVT) consisted of patients who received EVT. Cohort B (IVT-LVO) consisted of patients receiving IVT alone with Large Vessel Occlusion. LVO was defined as occlusion of the internal carotid artery (ICA) and M1 segment of Middle Cerebral Artery (MCA) only. Cohort C (IVT-nonLVO) consisted of IVT only patients without LVO, including MCA occlusions beyond M1, anterior cerebral artery occlusions (and/or CTP patterns consistent with distal occlusions not easily visualized on CTA).

### Imaging Acquisition and Analysis

All patients underwent pre-treatment multimodal CT and 24-h MRI or multimodal CT (if MR-incompatible) (10).

All CTP were post-processed with MIStar (Apollo Medical Imaging Technology, Melbourne, Australia) with both standard Single Value Deconvolution (sSVD, which is delay-sensitive), and also by delay and dispersion corrected Single Value Deconvolution (ddSVD, which is delay-insensitive) (11, 12). The software automatically performs motion correction and selects an arterial input function (AIF) from an unaffected artery (most often the anterior cerebral artery). Then the AIF was confirmed by experienced analysts (C.C, a neuroscientist with >6 years experience of perfusion imaging; and A.B, a neuroscientist with >10 years experience). The sSVD method generates maps of: standard cerebral blood volume (CBV), standard CBF, standard MTT and Tmax. Tmax is calculated from the time to peak of the impulse residual function (IRF) curve, where Tmax=0 reflects normal blood supply in normal tissue without delay and dispersion. DT was calculated using ddSVD method to correct for the potential arterial delay and dispersion effects caused by stroke and arterial stenosis by generating an arterial transport function from each voxel IRF (13).

#### Threshold Setting to Define Perfusion Deficit and Ischemic Core

Dual threshold setting was used to define perfusion deficit and ischemic core, with upper threshold defining the perfusion deficit and lower threshold defining ischemic core. Three thresholds were used according to previously published thresholds to define perfusion deficit: (i) MTT >145% of contralateral normal tissue (derived from sSVD) (14), (ii) Tmax >6 s (derived from sSVD) (3, 4, 15), (iii) DT> 3 s (derived from ddSVD) (11, 16). The threshold of rCBF <30% was applied to measure ischemic core within each of the perfusion deficit defined by the above thresholds (17). Mismatch ratio was defined as the perfusion deficit divide by the infarct core volume; mismatch volume was defined as the perfusion deficit volume minus the ischemic core volume.

For the EVT cohort, recanalization status was graded by Thrombolysis in Cerebral Infarction (TICI) grading system post-procedure Digital Subtraction Angiography (DSA). For IVT patients, recanalization status was graded by comparing follow-up MRA/CTA to acute CTA, evaluating the restoration of the previously occluded artery with Thrombolysis in Myocardial Infarction scoring system. For this study, we classified recanalization status as either (i) recanalization = TICI 2b, 2c, or 3 on DSA or TIMI 3 on follow-up MRA/CTA, or (ii) no recanalization = TICI 0, 1, or 2a on DSA, or TIMI 0, 1, 2 on follow-up MRA/CT. Collateral supply to the mismatch area was classified as 1 = good, 2 = moderate, 3 = poor using the Miteff grading system (18).

### Mismatch Profile Definition

Each patient was then classified using 6 separate mismatch criteria according to previously used mismatch criteria using the following methods and thresholds:

#### Perfusion Imaging Mismatch Profile (PIM-DT/PIM-Tmax/PIM-MTT)

PIM – mismatch between perfusion deficit and ischemic core: Mismatch ratio >1.8, mismatch volume >15 ml, core volume <70 ml, as determined by 3 different measures to define the perfusion deficit (DT >3 s, Tmax >6 s, or MTT >145%), and ischemic core defined as rCBF <30% constrained to the territory of the perfusion deficit defined in three ways as above;

#### Clinical Core Mismatch Profile (CCM-DT/CCM-Tmax/CCM-MTT)

CCM - mismatch between age-adjusted NIHSS and CTP core: NIHSS ≥10 and ischemic core volume <31 ml (age <80), or NIHSS ≥20 and ischemic core volume 31–51 ml (age <80); or NIHSS ≥10 and ischemic core volume <21 ml (age ≥80); as ischemic core volume determined by rCBF<30% constrained to the territory of the perfusion deficit defined in three ways (DT >3 s, Tmax >6 s, orMTT >145%).

### Statistical Analysis

Descriptive results and quantitative baseline patient characteristics were presented as median and Interquartile Range (IQR). Comparisons of continuous variables between groups were performed with Wilcoxon rank-sum test. Categorical variables were presented as proportions. Categorical variables were compared by chi-square, or Fisher's exact test as appropriate. The proportions of patients selected by each mismatch criterion were compared groups 2 by 2 as assessed by McNemar Test for discordant pairs. In patients with the same mismatch profile, differences of outcome variables (rate of mRS0-1, rate of mRS0-2, sICH and mortality rate) were compared between patients with and without recanalization. Furthermore, in patients with the same recanalization status, differences of outcome variables were compared between patients with and without target mismatch. Separate univariate logistic regression was constructed to assess the relationship between recanalization and excellent outcome/good outcome in patients with and without target mismatch utilizing each mismatch criterion. This was followed by multiple logistic regressions adjusting for age, and baseline core volume.

All the statistical analyses were performed for 3 cohorts of patients separately. Significant level was set at *P* < 0.05. Statistical analyses were performed with STATA 13.0 (Stata Corp, College Station, Texas, USA).

## Results

**Figure 1:**
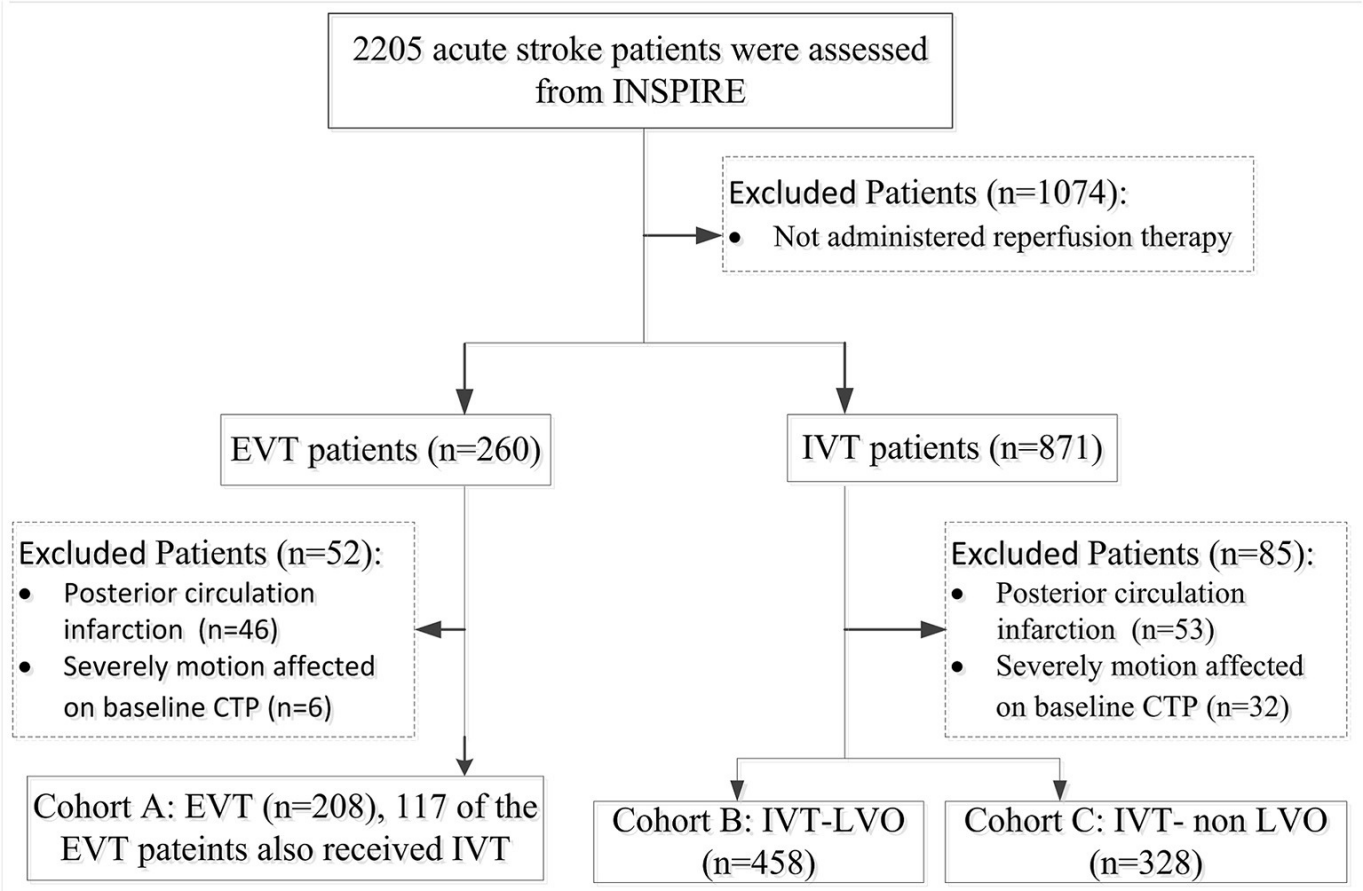
Flowchart of Patients Inclusion. INSPIRE, INternational Stroke Perfusion Imaging REgistry; EVT, endovascular Thrombectomy; IVT-LVO, Intravenous Thrombolysis patients with Large Vessel Occlusion; IVT-non-LVO, Intravenous Thrombolysis patients with no Large Vessel Occlusion, Vessel Occlusion.

During the study period, a total of 2,205 patients were enrolled in INSPIRE. A total of 994 patients were eligible for this study after various exclusions (patient inclusion was detailed in Figure 1). Cohort A consisted of 208 EVT patients (147 of the 208 EVT patients also received IVT); Cohort B consisted of 458 IVT-LVO patients; Cohort C consisted of 328 IVT-non LVO patients. Patients without an LVO had smaller baseline perfusion lesion, greater likelihood of good collaterals and a higher rate of excellent outcome (mRS 0-1 ate day-90) compared with patients with an LVO treated with EVT and/or IVT (Table 1). In patients with an LVO, EVT resulted in a higher rate of recanalization compared to IVT alone (78 vs. 47%, Table 1).

**Table 1:** Clinical and imaging characteristics.

**Parameter**	**EVT (*n* = 208)**	**IVT-LVO (*n* = 458)**	**IVT-non-LVO (*n* = 328)**
Age, median (IQR)	70 (59, 78)	73 (64, 81)	71 (60, 82)
Sex (male %)	59	53	60
Baseline NIHSS median (IQR)	15 (11-19)	15 (12-18)	8 (6-12)
24 h NIHSS median (IQR)	10 (3-17)	11 (5-7)	3 (1-6)
Median 90-days mRS (IQR)	3 (1-4)	3 (1-5)	1 (0, 1)
mRS 0-1 at 90-days, (%)	35	30	61
mRS 0-2 at 90-days, (%)	44	42	74
Median baseline ischemic core (rCBF <30% within DT >3s) (IQR), mL	20 (9, 42)	20 (7, 40)	3 (1-40)
Median baseline perfusion deficit volume (DT >3 s) (IQR), mL	109 (66,154)	95 (55, 144)	17 (5, 44)
Median 24 h infarct volume (IQR), mL	39 (14, 94)	35 (12, 107)	3 (1-12)
Median onset to lysis time (IQR), minutes	148 (95, 255)	153 (82, 315)	155 (96,206)
Onset to recanalization time (EVT patients)	271 (109, 645)	–	–
**Occlusion location**			
ICA (%)	32	28	–
M1 (%)	57	72	–
M2 (%)	11	–	42
M3 (%)	–	–	15
ACA (%)	–	–	5
No visible occlusion (%)	–	–	38
Recanalization rate (%)	78	47	87
mRS 0-1 in patient with recanalization, (%)	57	55	67
mRS 0-1in patient without recanalization, (%)	15	6	3
sICH (%)	5	4	1

*EVT, endovascular Thrombectomy; IVT-LVO, Intravenous Thrombolysis patients with Large Vessel Occlusion; IVT-nonLVO, Intravenous Thrombolysis patients without Large Vessel Occlusion; IQR, interquartile range; NIHSS, National Institutes of Health Stroke Score; mRS, modified Rankin Scale; rCBF, relative cerebral blood flow; DT, delay time; M1, M1segment of middle cerebral artery; M2, M2 segment of middle cerebral artery; ICA, internal carotid artery; sICH, symptomatic intracranial hemorrhage*.

### EVT Cohort

**Figure 2:**
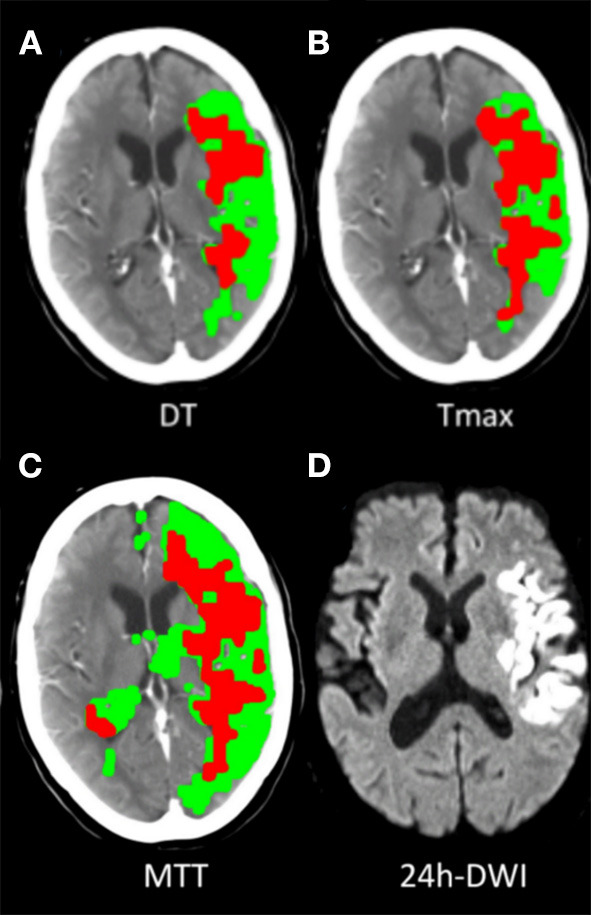
Illustrative examples of target mismatch classified by 3 PIM criteria. A 67-year-old case, acute left middle cerebral artery (MCA) M1 segment occlusion with sudden onset of right-side hemiparesis (baseline NIHSS 24), underwent endovascular thrombectomy, had successful recanalization (TICI 2c) and had mRS 1 at 90-day. **(A)** Classified as target mismatch by PIM-DT, with mismatch ratio 2.2, perfusion deficit volume 119 mL, CTP core volume 55 mL (defined as DT >3S and rCBF<30%). **(B)** Classified as non-target mismatch by PIM-Tmax, with mismatch ratio 1.5, perfusion deficit volume 116 mL, CTP core volume 78 mL (defined as Tmax >6S and rCBF<30%). **(C)** Classified as non-target mismatch by PIM-MTT, with mismatch ratio 1.8, perfusion deficit volume 166 mL, CTP core volume 98 mL (defined as MTT >145% and rCBF<30%). **(D)** 24-h DWI detected 46 mL infarct core.

Of the patients treated with EVT, 82.7% (172/208) met the PIM-DT criterion, which had the highest proportion of eligible patients. The proportions of patients selected by each mismatch criterion were significantly lower when compared with PIM-DT (Table 2, illustrative example of the disagreement between mismatch criteria in Figure 2). Between 32% (66/208 from CCM-DT) and 61% (127/208 from CCM-MTT) of patients were excluded due to the age-adjusted NIHSS/core cut off when using the CCM criteria. A total of 28% (58/208) of the patients were excluded due to the large core (infarct core volume ≥70 mL) when assessed with PIM-MTT, 20% (42/208) when assessed with PIM-Tmax, and 12% (24/208) when assessed with PIM-DT (Table 2).

**Table 2:** Disagreement between mismatch criteria and detail of exclusion.

			**Number of patients were excluded by each reason, n (%)**				
	**Mismatch (+), n (%)**	***P*-value**	**Large core**	**Small penumbra**	**Mismatch ratio <1.8**	**Age/core cut off**	**Low NIHSS**
**EVT (*****n*** **= 208)**							
PIM-DT	172 (82.7)	–	24 11.5)	12 (5.8)	0 (0)	–	–
PIM-Tmax	142 (68.3)	<0.0001	42 (20.2)	18 (8.7)	0 (0)	–	–
PIM-MTT	149 (71.6)	0.0023	58 (27.9)	1 (0.5)	0 (0)	–	–
CCM-DT	114 (54.8)	<0.0001	–	–	–	66 (31.7)	28 (13.5)
CCM-Tmax	74 (35.6)	<0.0001	–	–	–	106 (51.0)	28 (13.5)
CCM-MTT	53 (25.5)	<0.0001	–	–	–	127 (61.1)	28 (13.5)
**IVT-LVO (*****n*** **= 458)**							
PIM-DT	364 (79.5)	–	49 (10.7)	5 (1.1)	1 (0.2)	-	-
PIM-Tmax	310 (67.7)	<0.0001	76 (16.6)	45 (9.8)	27 (5.9)	-	-
PIM-MTT	333 (72.7)	0.0003	118 (25.8)	5 (1.1)	1 (0.2)	-	-
CCM-DT	211 (46.1)	<0.0001	–	–	–	162 (35.4)	84 (18.3)
CCM-Tmax	174 (38.0)	<0.0001	–	–	–	200 (43.7)	84 (18.3)
CCM-MTT	128 (27.9)	<0.0001	–	–	–	246 (53.7)	84 (18.3)
**IVT-nonLVO (*****n*** **= 328)**							
PIM-DT	152 (46.3)	<0.0001	1(0.3)	173 (52.7)	2 (0.6)	–	–
PIM-Tmax	120 (36.6)	<0.0001	5(1.5)	191 (58.2)	12 (3.7)	–	–
PIM-MTT	249 (75.9)	–	12 (3.7)	61 (18.3)	6 (1.8)	–	–
CCM-DT	117 (35.7)	<0.0001	–	–	–	11 (3.3)	200 (61.0)
CCM-Tmax	105 (32.0)	<0.0001	–	–	–	23 (7.0)	200 (61.0)
CCM-MTT	82 (25.0)	<0.0001	–	–	–	46 (1.4)	200 (61.0)

*EVT, endovascular Thrombectomy; IVT-LVO, Intravenous Thrombolysis patients with Large Vessel Occlusion; IVT-nonLVO, Intravenous Thrombolysis patients without Large Vessel Occlusion; PIM, Perfusion Imaging Mismatch; CCM, Clinical Core Mismatch; Significant level was set at P < 0.05, when the proportion of each mismatch criteria compared with the mismatch criteria had the highest proportion in each patient cohort, separately*.

Recanalization was strongly associated with achieving an excellent outcome and good outcome at 90-days in patients meeting the PIM (DT/Tmax/MTT) criteria (e.g., PIM-DT + patients, mRS 0-1 adjusted OR: 4.27 95% CI: 1.53, 11.91, *P* = 0.005, Tables 3A,B). Whereas, there was no such association between recanalization and excellent or good outcome at 90-days in target mismatch patients classified by any of the CCM criteria (Tables 3A,B). Additionally, patients meeting the PIM (DT/Tmax/MTT) mismatch criteria had a higher rate of excellent outcome after recanalization (e.g., PIM-DT+ patient, 43.4%, 59/136 with recanalization, vs. 13.9%, 5/36 without recanalization, *P* = 0.001, Tables 4A,B). Importantly, PIM-DT was the only mismatch criterion that showed target mismatch patients had a higher rate of excellent or good outcome compared with non-target mismatch patients after recanalization (e.g., 43.4%, 59/136 PIM-DT+ patients with recanalization vs. 26.9%, 7/26 PIM-DT- patients with recanalization, *P* = 0.013, Table 4A).

**TABLE 3A:** The relationship between mismatch predicting mRS 0-1 in patients with recanalization.

	**EVT**		**IVT-LVO**		**IVT-nonLVO**	
**Mismatch**	**Adjusted OR (95%CI)**	***P***	**Adjusted OR (95%CI)**	***P***	**Adjusted OR (95%CI)**	***P***
**PIM-DT**						
yes	4.27 (1.53, 11.91)	0.005	9.77 (4.83, 19.78)	<0.0001	4.91 (1.34, 17.97)	0.016
no	1.01 (0.14, 7.13)	0.992	9.32 (2.37, 35.90)	0.001	4.79 (1.38, 16.65)	0.014
**PIM-Tmax**						
yes	4.37 (1.58, 12.12)	0.025	14.39 (5.32, 38.93)	<0.0001	4.75 (1.03, 21.84)	0.045
no	3.25 (0.34, 31.07)	0.306	8.99 (4.02, 20.10)	<0.0001	7.52 (2.32, 24.29)	0.001
**PIM-MTT**						
Yes	5.66 (1.75, 18.26)	0.004	9.93 (4.96, 19.86)	<0.0001	5.01 (1.93, 13.03)	0.001
No	1.99 (0.41, 9.65)	0.394	22.03 (4.78, 101.45)	<0.0001	7.64 (0.82, 70.52)	0.043
**CCM-DT**						
Yes	5.74 (1.22, 27.02)	0.027	11.16 (4.95, 15.23)	<0.0001	6.15 (1.56, 24.24)	0.009
No	3.78 (1.28, 11.19)	0.016	7.96 (3.06, 20.71)	<0.0001	3.12 (0.94, 10.40)	0.044
**CCM-Tmax**						
Yes	6.24 (0.62, 62.88)	0.120	11.17 (4.62, 26.99)	<0.0001	5.69 (1.36, 23.94)	0.018
No	3.73 (1.34, 10.38)	0.012	10.35 (4.34, 24.68)	<0.0001	4.37 (1.42, 13.580)	0.011
**CCM-MTT**						
Yes	4.56 (1.75, 11.94)	0.310	9.73 (3.49, 27.14)	<0.0001	6.86 (1.25, 37.38)	0.026
No	5.43 (2.12, 13.93)	0.460	11.99 (5.55, 25.90)	<0.0001	4.74 (1.67, 13.41)	0.003

*mRS 0-1, modified Rankin Score 0-1 at 90-days; EVT, endovascular Thrombectomy; IVT-LVO, Intravenous Thrombolysis patients with Large Vessel Occlusion; IVT-nonLVO, Intravenous Thrombolysis patients without Large Vessel Occlusion; PIM, Perfusion Imaging Mismatch; CCM, Clinical Core Mismatch*.

**TABLE 3B:** The relationship between mismatch predicting mRS 0-2 in patients with recanalization.

	**EVT**		**IVT-LVO**		**IVT-nonLVO**	
**Mismatch**	**Adjusted OR (95%CI)**	***P***	**Adjusted OR (95%CI)**	***P***	**Adjusted OR (95%CI)**	***P***
**PIM-DT**						
Yes	6.69 (2.42, 18.50)	<0.0001	12.67 (7.41, 21.67)	<0.0001	2.69 (0.93, 7.73)	0.067
No	1.88 (0.29, 12.12)	0.503	5.15 (1.41, 18.79)	<0.0001	6.99 (1.54, 31.61)	0.012
**PIM-Tmax**						
Yes	5.90 (2.17, 16.04)	<0.0001	11.15 (6.34, 19.58)	<0.0001	2.04 (0.63, 6.66)	0.234
No	6.12 (0.69, 54.13)	0.103	13.48 (5.30, 34.25)	<0.0001	7.48 (2.29, 24.38)	0.001
**PIM-MTT**						
Yes	7.17 (2.27, 22.69)	0.001	12.04 (7.00, 20.69)	<0.0001	4.47 (1.86, 10.76)	0.001
No	4.10 (0.94, 17.98)	0.061	12.78 (4.60, 35.49)	<0.0001	2.72 (0.40, 18.37)	0.303
**CCM-DT**						
Yes	9.10 (1.96, 42.25)	0.005	10.72 (5.46, 21.03)	<0.0001	7.28 (2.14, 24.72)	0.001
No	3.86 (1.20, 12.39)	0.023	11.87 (5.71, 24.67)	<0.0001	1.47 (0.42, 5.16)	0.546
**CCM-Tmax**						
Yes	8.67 (0.94, 80.06)	0.057	9.96 (4.83, 20.52)	<0.0001	7.18 (1.94, 26.58)	0.003
No	6.05 (2.19, 16.68)	0.001	13.68 (7.11, 26.32)	<0.0001	2.13 (0.71, 6.4)	0.175
**CCM-MTT**						
Yes	2.73 (0.27, 27,99)	0.396	10.62 (4.48, 25.14)	<0.0001	7.80 (1.72, 35.4)	0.008
No	7.31 (2.79, 19.11)	<0.0001	13.14 (7.39, 23.39)	<0.0001	2.99 (1.12, 7.99)	0.029

*mRS 0-2, modified Rankin Score 0, 1, 2 at 90-days; EVT, endovascular Thrombectomy; IVT-LVO, Intravenous Thrombolysis patients with Large Vessel Occlusion; IVT-nonLVO, Intravenous Thrombolysis patients without Large Vessel Occlusion; PIM, Perfusion Imaging Mismatch; CCM, Clinical Core Mismatch*.

**TABLE 4A:** Outcomes based on target mismatch profile and recanalization status.

		**EVT**			**IVT-LVO**			**IVT-nonLVO**		
**Mismatch**	**RECAN**	**mRS 0-1 (%)**	**Mortality rate (%)**	**sICH (%)**	**mRS 0-1 (%)**	**Mortality rate (%)**	**sICH (%)**	**mRS 0-1 (%)**	**Mortality rate (%)**	**sICH (%)**
**PIM-DT**										
Yes	Yes	43.4^*^	9.6^*^	3.7	58.2^*^	3.21^*^	1.0^*^	63.3^*^	1.1	1.4
Yes	No	13.9	30.6	5.6	7.7	21.5	7.7	28.6	0.0	0.0
	***P***	0.001	0.003	0.451	<0.0001	<0.0001	0.006	0.019	0.952	0.946
No	Yes	26.9	26.9	11.5	31.0^*^	11.4^*^	7.7	62.1^*^	4.8	2.4
No	No	20.0	50.0	0	3.1	34.2	5.8	22.2	16.7	6.7
	***P***	0.514	0.178	0.364	<0.0001	<0.0001	0.701	0.002	0.088	0.393
**PIM-Tmax**										
Yes	Yes	46.5^*^	8.2^*^	3.6	57.8^*^	3.7^*^	2.3	61.1	0.0	0
Yes	No	18.8	28.1	6.2	7.3	20.6	7.4	39.4	9.1	0
	***P***	0.005	0.006	0.408	<0.0001	<0.0001	0.148	0.184	0.169	1
No	Yes	28.9	21.2^*^	7.7	44.2^*^	6.3^*^	3.8	63.1^*^	4.4	2.7
No	No	7.1	50.0	0	5.2	30.5	6.9	19.1	9.5	5.9
	***P***	0.085	0.045	0.376	<0.0001	<0.0001	0.371	<0.0001	0.28	0.433
**PIM-MTT**										
Yes	Yes	42.2^*^	10.7^*^	4.1	58.1^*^	3.3^*^	4.1	65.1^*^	3.5	1.6
Yes	No	14.3	32.1	7.1	8.4	18.2	7.7	29.2	4.2	3.8
	***P***	0.004	0.008	0.391	<0.0001	<0.0001	0.254	0.001	0.918	0.336
No	Yes	36.6	17.1	7.3	41.3^*^	9.8^*^	0	53.4^*^	2.4	3.3
No	No	16.7	38.9	0	2.6	40.0	6.2	12.5	25.0	0
	***P***	0.109	0.072	0.328	<0.0001	<0.0001	0.101	0.045	0.063	0.981
**CCM-DT**										
Yes	Yes	35.8^*^	10.5	4.3	61.9^*^	3.6^*^	4.2	53.6^*^	6.6	0
Yes	No	10.6	26.3	5.3	9.7	14.7	3.1	17.6	11.7	0
	***P***	0.031	0.075	0.605	<0.0001	<0.0001	0.734	0.008	0.608	1
No	Yes	46.3^*^	14.9^*^	6.0	46.7^*^	6.7^*^	1.5^*^	67.4^*^	1.5	2.9
No	No	18.5	40.7	3.7	4.3	35.1	9.8	33.3	6.7	8.3
	***P***	0.018	0.012	0.553	<0.0001	<0.0001	0.03	0.009	0.268	0.366
**CCM-Tmax**										
Yes	Yes	30.8	12.3	4.6	62.2^*^	3.2^*^	3.8	52.2^*^	5.9	0
Yes	No	11.1	33.3	11.1	10.5	17.7	3.9	20.0	13.3	0
	***P***	0.209	0.125	0.412	<0.0001	0.002	0.971	0.043	0.301	1
No	Yes	47.4^*^	12.4^*^	5.2	48.8^*^	6.4^*^	2.3	67.4^*^	2.0	2.8
No	No	16.2	35.1	2.7	4.5	31.5	8.6	29.4	5.9	7.1
	***P***	0.001	0.005	0.270	<0.0001	<0.0001	0.065	0.002	0.357	0.383
**CCM-MTT**										
Yes	Yes	22.9	14.6	2.1	57.8^*^	2.9^*^	3.5	52.9^*^	3.9	0
Yes	No	20.0	40.0	0	10.5	21.3	5.1	16.7	8.3	0
	***P***	0.685	0.196	0.906	<0.0001	<0.0001	0.697	0.028	0.476	1
No	Yes	48.3^*^	11.4^*^	6.1	53.3^*^	5.9^*^	2.8	65.6^*^	3.1	2.5
No	No	14.6	34.2	4.9	5.1	28.8	7.7	30.0	10.0	6.2
	***P***	<0.0001	0.003	0.559	<0.0001	<0.0001	0.101	0.002	0.171	0.402

*RECEN, recanalization; sICH, symptomatic intracerebral haemorrhagic; EVT, endovascular Thrombectomy; IVT-LVO, Intravenous Thrombolysis patients with Large Vessel Occlusion; IVT-nonLVO, Intravenous Thrombolysis patients without Large Vessel Occlusion; PIM, Perfusion Imaging Mismatch; CCM, Clinical Core Mismatch*.

* *Denote a significant difference present when compared with patients with the same mismatch profile, but without recanalization*.

**TABLE 4B:** Outcomes based on target mismatch profile and recanalization status.

		**EVT**	**IVT-LVO**	**IVT-nonLVO**
**Mismatch**	**Recanalization**	**mRS0-2 (%)**	**mRS0-2 (%)**	**mRS0-2 (%)**
**PIM-DT**				
Yes	Yes	54.4^*^	72.7^*^	76.7
Yes	No	13.9	17.9	57.1
	***P***	<0.0001	<0.0001	0.061
No	Yes	42.3	51.7^*^	77.5^*^
No	No	20.0	7.7	27.3
	***P***	0.197	<0.0001	0.001
**PIM-Tmax**				
Yes	Yes	58.2^*^	72.7^*^	78.4^*^
Yes	No	18.8	19.9	31.3
	***P***	<0.0001	<0.0001	<0.0001
No	Yes	40.4^*^	65.4^*^	75.0
No	No	7.1	9.4	62.5
	***P***	0.016	<0.0001	0.232
**PIM-MTT**				
Yes	Yes	53.7^*^	72.6^*^	77.9^*^
Yes	No	14.3	18.2	46.2
	***P***	<0.0001	<0.0001	0.001
No	Yes	48.8^*^	60.9^*^	72.7
No	No	16.7	8.9	50.0
	***P***	0.018	<0.0001	0.257
**CCM-DT**				
Yes	Yes	49.5^*^	75.4^*^	75^*^
Yes	No	10.5	23.7	35.3
	***P***	0.001	<0.0001	0.003
No	Yes	56.7^*^	64.5^*^	78.3
No	No	18.5	9.3	60.0
	***P***	0.001	<0.0001	0.107
**CCM-Tmax**				
Yes	Yes	44.6	74.5^*^	73.1^*^
Yes	No	11.1	23.7	33.3
	***P***	0.055	<0.0001	0.005
No	Yes	57.7^*^	66.9^*^	78.9
No	No	16.2	10.8	58.8
	***P***	<0.0001	<0.0001	0.065
**CCM-MTT**				
Yes	Yes	37.5	70.1^*^	72.6^*^
Yes	No	20.0	13.6	33.3
	***P***	0.404	<0.0001	0.014
No	Yes	58.8^*^	70.4^*^	78.5^*^
No	No	14.6	19.3	55
	*P*	<0.0001	<0.0001	0.025

*EVT, endovascular Thrombectomy; IVT-LVO, Intravenous Thrombolysis patients with Large Vessel Occlusion; IVT-nonLVO, Intravenous Thrombolysis patients without Large Vessel Occlusion; PIM, Perfusion Imaging Mismatch; CCM, Clinical Core Mismatch*.

* *Denote a significant difference present when compared with patients with the same mismatch profile, but without recanalization*.

### IVT-LVO Cohort

In the cohort of LVO patients treated with IVT only, 79.5% (364/458) met the PIM–DT, which had the highest proportion of eligible patients. The proportions of patients selected by the other mismatch criteria were also significantly lower than PIM-DT (Table 2). When using the CCM criteria, between 35% (162/458 from CCM-DT) and 54% (246/458 from CCM-MTT) of the patients were excluded due to the age-adjusted NIHSS/core cut off. A total of 28% (118/458) of the patients were excluded due to large core (core volume ≥70 mL) when assessing with PIM-MTT, whilst 17% (76/458) when assessing with PIM-Tmax and 11% (49/458) using PIM-DT criterion (Table 2).

In contrast to the EVT cohort, recanalization was associated with an excellent and good outcome at day-90 in patients with and without target mismatch regardless of the type of mismatch criteria used (Tables 3A,B). The target mismatch patients with recanalization had a higher rate of excellent and good outcome compared with target mismatch patients without recanalization, regardless of the type of mismatch criteria used (e.g., rate of mRS 0-1: 58.2% in PIM-DT+ with recanalization vs. 7.7% in PIM-DT+ without recanalization, *P* < 0.0001, Tables 4A,B). A similar relationship was also seen in non-target mismatch patients (e.g., rate of mRS 0-1: 31.0% in PIMDT- with recanalization vs. 3.1% in PIM-DT- without recanalization, *P* < 0.0001, Tables 4A,B).

However, PIM-DT was the only mismatch criterion that showed target mismatch patients had a higher rate of excellent outcome compared with non-target mismatch patients after recanalization. A total of 58.2 % (114/196) PIM-DT+ patients achieved an excellent outcome at day-90 after recanalization, compared with only 31.0% (9/29) in PIM-DT- patients (*P* = 0.006, Table 4A). Furthermore, PIM-DT was again the only criterion that showed a reduced rate of sICH in PIM-DT+ patients with recanalization (1%, 2/196) compared with PIM-DT+ patients without (7.7%, 13/168, *P* = 0.006, Table 4A).

### IVT-Non-LVO Cohort

In the cohort of IVT patients without an LVO, 75.9% (249/328) met the PIM-MTT criterion, which had the highest proportion of eligible patients. The proportions of patients selected by each mismatch profile were significantly smaller when compared with PIM-MTT (Table 2). The majority of non-target mismatch patients (61%, 200/328) were excluded due to low NIHSS when using the CCM criteria. A significant number of patients (19%, 61/328 from PIM-MTT; 58%, 191/328 from PIM-Tmax; and 53%, 173/328 from PIM-DT) were classified as non-mismatch due to small penumbral volume (≤ 15 mL, Table 2).

Recanalization was associated with excellent and good outcome in patients with and without target mismatch regardless of mismatch criteria (Tables 3A,B). In IVT patients without an LVO, the adjusted OR of excellent outcome after recanalization (compared to no recanalization) in PIM-MTT+ patients was 5.01 (95% CI 1.93, 13.03, *P* = 0.001 Table 3), whereas in PIM-MTT- patients the adjusted OR of an excellent clinical outcome with recanalization was 7.64 (95% CI 0.82, 70.52, *P* = 0.043, Table 2). Mortality and sICH were not associated with recanalization in patients with and without target mismatch regardless of the type of mismatch criteria (Table 4A). There was no significant difference in the percentage of an excellent or good outcome at day-90, between target mismatch and non-target mismatch patients with recanalization, regardless of the type of mismatch criteria used (Tables 4A,B).

## Discussion

This study assessed a range of PIM and CCM criteria in a large ischemic stroke cohort from INSPIRE. It demonstrated that PIM-DT was the optimal target mismatch criterion to identify “excellent” responders to recanalization for LVO patients, treated with either EVT or IVT. The PIM-DT criterion had the highest proportion of eligible target mismatch patients and was the only of the six criteria to distinguish responders from non-responder to reperfusion in LVO patients.

For LVO patients receiving EVT and/or IVT, the CCM criterion was a more restrictive selection criterion compared to the PIM criteria regardless of the post-processing method used to define perfusion deficit and ischemic core. A large number of patients (32% to 61%) were excluded due to the age-adjusted NIHSS/core cut off. The strict age-adjusted NIHSS/core cut-off excluded patients with a relatively small ischemic core volume who might well-still benefit from reperfusion therapy (19). This is highly relevant to everyday practice and individual patient since the rates of excellent or good outcome after recanalization were similar between patients with and without CCM.

Both the DAWN (1) and DEFUSE 3 (3) trials demonstrated significant treatment benefits of thrombectomy extending to a later time window, despite different target mismatch criteria being applied. Clinical core mismatch was used in the DAWN trial, which has strict age-adjusted NIHSS/pre-treatment core volume cut off. In contrast, the DEFUSE 3 trial required perfusion imaging mismatch, a discrepancy between penumbra and ischemic core. However, patients who were excluded from the clinical core mismatch (due to large pre-treatment core) but met the perfusion imaging mismatch were shown to still benefit from reperfusion treatment (19). These two different patient selection techniques produced similar trial results which resulted in significant global practice change. However, it is clear that there is some refinement that can be done, where patients at the peripherals or even just edging into exclusion may benefit from treatment, but to less of an extent to those who are eligible. The challenge will be to definitely identify where the futility margin exists, and perhaps where harm even starts. Compounding this challenge is the issue that the thresholds to measure penumbra and core vary from different post-processing algorithms or software (11) which result in large discrepancies between vendors. The specific thresholds (Tmax > 6 s and rCBF<30%) used in the DEFUSE 3 and EXTEND-IA trial were calculated by sSVD, which are known to overestimate of the perfusion deficit (7, 8, 11). Without delay and dispersion correction, 15% of the patients who would potentially benefit for reperfusion treatment might be excluded when applying perfusion imaging mismatch criteria.

For patients without an LVO who received IVT, the PIM-MTT had a reasonable rate of eligibility, compared with other criteria. This may be due to the overestimation of the perfusion deficit that leads to a high rate of inclusion (20, 21). The MTT is less sensitive to spontaneous reperfusion as CBV may be increased more than CBF due to spontaneous reperfusion leading to prolonged MTT whereas DT and Tmax will be lower as they are direct measures of reperfusion (22). However, none of the mismatch criterion was able to identify the non-LVO patients who most likely benefit from reperfusion therapy, since there was no significant difference in the rate of excellent or good clinical outcomes between patient with and without mismatch after recanalization. It is likely that some patients in the IVT-non-LVO cohort with a distal perfusion deficit, but no clear vessel occlusion on CTA, might have been undergoing spontaneous recanalization and reperfusion before imaging. Thus, these patients may have begun with target mismatch but by the time of imaging were non-mismatch. The majority of the patients (87%) in non LVO group achieved recanalization. These groups of patients have a high rate of spontaneous reperfusion and hence will have a good clinical outcome with or without thrombolysis (23, 24), with the majority of the patients (70%) without an LVO having a baseline perfusion deficit of <15 mL.

Some limitations of the study need to be acknowledged. This is an observational study using data from INSPIRE, which is a large dataset collected from multiple sites. Whilst sites are strongly encouraged to recruit consecutive patients, this is not always possible and there may be recruitment biases which cannot be measured. In particular, the information about whether the mismatch criteria used in assisting decision-making was not available, there may be undocumented factors behind the treatment decision making. “Furthermore, there might be some unmeasured bedside bias influencing the reperfusion treatment decision making because of clinical judgment in case selection for reperfusion treatment remain variability. The current study only included anterior circulation ischemic stroke patients. The results are un-likely to be relevant to patients with posterior circulation ischemic stroke. It is important to acknowledge that our findings are specific to a particular post-processing imaging technique, and as such, our results might not be directly translated to other perfusion software currently used (25, 26). Nevertheless, the underlying principles of the algorithms (sSVD/ddSVD) using in different software are the same, we would expect that the PIM calculated with delay insensitive method would be the optimal target mismatch criterion that can identify patients with LVO most likely response to reperfusion therapy. Moreover, we used perfusion imaging from different scanners, which might slightly influence the results of imaging analysis. We assessed each of the mismatch criteria in the same patient cohort to reduce the influence from using perfusion imaging from different scanners.

In conclusion, the PIM-DT was the optimal target mismatch criterion to identify LVO patients most likely to have an excellent response to EVT and/or IVT. PIM-DT combined a relatively high rate of eligibility with high rates of response to recanalization (and with less sICH). In contrast, none of the mismatch criteria was useful to identify recanalization responders in non-LVO patients.

## Data Availability Statement

Anonymized data that support the findings of this study are available from the corresponding author upon reasonable request.

## Ethics Statement

The studies involving human participants were reviewed and approved by Hunter New England Health District ethics committees. The patients/participants provided their written informed consent to participate in this study.

## Author Contributions

CC, MP, and AB: study design, acquisition, analysis and interpretation of data, drafted the manuscript, revised it critically for important intellectual content, and approved the final version. CL, NS, LL, TK, KB, RA, and MK: acquisition and interpretation of data, revised the manuscript critically for important intellectual content, and approved the final version. XC, QD, BO'B, PS, PC, SB, CY, JY, PW, and WQ: data acquisition, revised the manuscript critically for important intellectual content, and approved the final version.

## Conflict of Interest

The authors declare that the research was conducted in the absence of any commercial or financial relationships that could be construed as a potential conflict of interest.
